# Supplementary material for: In-Depth Phenotyping of PIGW-Related Disease and Its Role in 17q12 Genomic Disorder
Source: Biomolecules. 2024 Dec 18;14(12):1626. doi: 10.3390/biom14121626 (PMC11727550; doi:10.3390/biom14121626)
Supplement: Supplementary file 1 [file biomolecules-14-01626-s001.zip › biomolecules-3203901-supplementary.pdf]

Supplemental data

In-Depth Phenotyping of *PIGW*-Related Disease and Its Role in 17q12 Genomic Disorder

Agnese Feresin [1,2,*], Mathilde Lefebvre [2,3], Emilie Sjøstrøm [4], Caterina Zanus [5], Elisa Paccagnella [5], Irene Bruno [5], Erica Valencic [5], Anna Morgan [5], Alberto Tommasini [1,5], Christel Thauvin [6], Allan Bayat [4,7,8,9], Giorgia Girotto [1,5] and Luciana Musante [5,*]

[1] Department of Medicine, Surgery and Health Sciences, University of Trieste, 34127 Trieste, Italy; alberto.tommasini@burlo.trieste.it (A.T.); giorgia.girotto@burlo.trieste.it (G.G.)
[2] SoFFoet, Société Française de Fœtopathologie, 75015 Paris, France; mathilde.becmeur-lefebvre@chu-orleans.fr
[3] UF de Foetopathologie, CHU d'Orléans, 45100 Orleans, France
[4] Department of Epilepsy Genetics and Personalized Medicine, Danish Epilepsy Center, 4293 Dianalund, Denmark; emiliebangsjostrom@gmail.com (E.S.); abaya@filadelfia.dk (A.B.)
[5] Institute for Maternal and Child Health, IRCCS "Burlo Garofolo", 34137 Trieste, Italy; caterina.zanus@burlo.trieste.it (C.Z.); elisa.paccagnella@burlo.trieste.it (E.P.); irene.bruno@burlo.trieste.it (I.B.); erica.valencic@burlo.trieste.it (E.V.); anna.morgan@burlo.trieste.it (A.M.)
[6] Inserm—UB UMR 1231 GAD "Génétique des Anomalies du Développement", Fédération Hospitalo-Universitaire-TRANSLAD, Dijon, France; christel.thauvin@chu-dijon.fr
[7] Department of Regional Health Research, University of Southern Denmark, 5230 Odense, Denmark
[8] Department of Drug Design and Pharmacology, University of Copenhagen, 2100 Copenhagen, Denmark
[9] Department of Paediatrics, Danish Epilepsy Center, 4293 Dianalund, Denmark
* Correspondence: feresin.agnese@gmail.com (A.F.); luciana.musante@burlo.trieste.it (L.M.)

Methods

WES analysis and interpretation

Patient 1: We extracted genomic DNA from venous peripheral blood using the QIAsymphony® SP instrument with the QIAsymphony® DNA Midi (Qiagen, Hilden, Germany) and processed DNA for library enrichment with the Twist Exome 2.0 plus Comprehensive Exome Spike-in (Twist Bioscience, San Francisco, CA, USA), according to the manufacturers' instructions. We executed sequencing on the NextSeq 500 (Illumina, San Diego, USA) instrument. Data processing, including sequence alignment to GRCh37/hg19, variant filtering and prioritization, were performed as previously reported [1]. In brief, sequencing reads were aligned to the human reference genome (GRCh37/hg19) employing the BWA-mem tool [2], and variant calling was performed with GATK v4.1.2 HaplotypeCaller (EnGenome, Pavia, Italy). We annotated and prioritized the variants with eVAI-enGenome software, based on the American College of Medical Genetics and Genomics (ACMG) guidelines [3]. SNVs and INDELs were filtered referring to public databases (dbSNP build150, Genome Aggregation Database (gnomAD v4.0)), which led to ruling out those variants previously reported as polymorphism. In particular, a minor allele frequency (MAF) cut-off of ≤0.01% was utilized. We examined the trio for the following inheritance patterns: *de novo*, homozygous recessive, and compound heterozygous. We also assessed the variant's pathogenicity with consultation of the ClinVar (https://www.ncbi.nlm.nih.gov/clinvar/), the Human Gene Mutation Database professional (HGMD) (Qiagen), Online Mendelian Inheritance in Man (OMIM) (https://www.omim.org/) and DECIPHER (https://www.deciphergenomics.org). Several *in silico* tools, such as PaPI [4], PolyPhen-2 [5], Sorting Intolerant from Tolerant (SIFT) [6], dbscSNV score [7] and Combined Annotation Dependent Depletion (CADD) score [8] were applied to establish the pathogenicity of novel variants. The diagnostic procedure adopted included discussing WES data in the context of phenotypic data at interdisciplinary meetings; systematic bibliographic review and public database consultation supported the interpretation of sequencing data.

Patient 2: We extracted DNA from peripheral blood using standard procedures and performed library preparation and enrichment for WES on PBL DNA following the standard Twist procedure for the Twist Library Preparation EF Kit 1.0, Enzymatic Fragmentation, Twist Universal Adapter System, and Twist Human Core Exome Kit (Twist Bioscience, San Francisco, CA, USA). The TWIST libraries were loaded to an S1 flow cell and sequenced in a paired-end 110 cycles on a NovaSeq 6000 system (Illumina, Inc., San Diego, CA, USA). We internally validated the NGS method and the variant analysis pipeline by analyzing five control samples per every 350 samples and externally by participating in the EMQN program. Data processing, including sequence alignment to GRCh37/hg19, variant filtering and prioritisation, were performed as previously reported [9]. In brief, we analyzed the possible modes of inheritance (sporadic de novo, dominant, recessive, X-linked) employing sensible minor population allele frequency cutoffs $\leq$ 0.01% in the Genome Aggregation Database (gnomAD v4.0). We evaluated genetic nonsynonymous missense, inframe insertions, inframe deletions, frameshift, stop_lost, stop_grain, 5-prime UTR (< 10 base pair upstream), splice site variants through database searches such as dbSNP155, ClinVar (https://www.ncbi.nlm.nih.gov/clinvar/), the Exome Aggregation Consortium database (ExAC), gnomAD, and Human Gene Mutation Database Professional (HGMD) (Qiagen). Prediction software such as PolyPhen2 [5], SIFT [6], MutationTaster [9] and CADD [8] were used to infer the pathogenicity of missense variants, while the PWM, MaxEntScan, GeneSplicer, and NNSplice prediction tools evaluated splice site variants. Variants analyzed under a dominant inheritance model observed more than three times in public databases (ExAC and gnomAD) were considered too common and discarded. We validated potentially pathogenic variants through conventional Sanger sequencing.

Patients 3 and 4: We extracted DNA from frozen tissues of the fetus and peripheral blood samples of both parents, using the QIAamp DNA Blood Mini Kit (Qiagen) following standard procedures. Whole-exome capture and sequencing were performed at Integragen SA from 1 µg of genomic DNA per individual using the SureSelect Human All Exon V5 kit (Agilent). We sequenced the resulting libraries on a HiSeq 2000 (Illumina) according to the manufacturer's recommendations for paired-end 76 bp reads and accomplished data processing, including sequence alignment to GRCh37/hg19, variant filtering and prioritization, as previously reported [11]. In brief, reads were aligned to the human genome reference sequence (GRCh37/hg19 build of UCSC Genome Browser) with the Burrows-Wheeler Aligner (BWA, v.0.6.2). We used the Genome Analysis Toolkit (GATK) v.2.6-4 for base quality score recalibration, indel realignment, and variant discovery (both single-nucleotide variants and indels) and registered them with SeattleSeq SNP Annotation. Rare variants were identified by focusing on protein-altering and splice-site changes present at a frequency of less than 1% in dbSNP 138 and Exome Aggregation Consortium (ExAC). The filtered variants' diagnostic interpretation was conducted according to local practices and following the ACMG recommendations 2015 [3]. We subjected the selected variants to thorough searches in the literature and databases and discussed each result multidisciplinary with the laboratory directors and the referring physicians. We systematically visualized candidate variants on the Integrative Genomic Viewer before fetal validation and familial segregation by Sanger sequencing for SNV.

Table S1: Summary of WES info and statistics

**Patient_ID**	**1**	**2**	**3**
**WES method and data processing (reference)**	PMID: 35670379 [1]	PMID: 35723786 [10]	PMID: 32732226 [11]
**WES approach**	Trio	Trio	Singleton
**Enrichment kit**	Twist Human Core for Enrichment-Exome panel (Twist Bioscience)	Twist Human Core Exome Kit (Twist Bioscience)	SureSelect Human All Exon V5 kit (Agilent)
**Sequencing platform**	NextSeq 500 (Illumina)	NovaSeq 6000 (Illumina)	HiSeq 2000 (Illumina)
**Target regions covered >10x**	98%	97%	97%
**Nucleotide changes (NM_178517.5)**	c.106A>G<br>c.1227T>G	c.50C>T<br>c.1202del	c.106A>G
**Amino acid changes (NP_848612)**	p.Arg36Gly<br>p.Cys409Trp	p.Thr17Ile<br>p.Leu401GlnfsTer6	p.Arg36Gly
**gnomAD**<br>**(status, allele frequency)**	het, 0.0004448<br>het, 0.00002169	not found<br>het, 0.00000186	het, 0.0004448
**ACMG classification** [3]	LP (PM2, PP2, PP3, PP5)<br>LP (PM2, PM3, PP2, PP3)	LP (PM2, PM3, PP1, PP2, PP3)<br>P (PVS1, PM2, PM4, PP1, PP3)	LP (PM2, PP2, PP3, PP5)

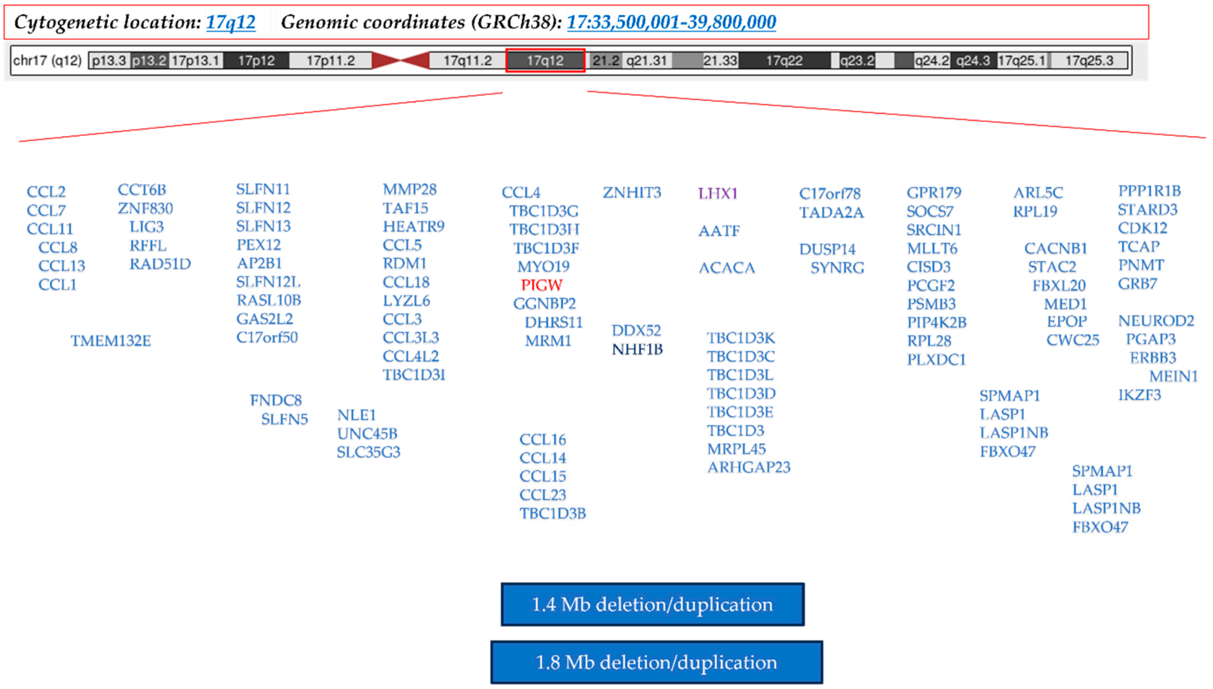

Figure S1. 17q12 genomic region. Representation of the genomic region 17q12 (Genomic coordinates (GRCh38): 17:33,500,001-39,800,000), showing major genes, from UCSC Genome Browser on Human (https://genome.ucsc.edu/index.html); *PIGW* is marked in red, *NHFB1* and *LHX1* are highlighted in dark blue and purple, respectively. Squares corresponding to the common recurrent deletions and duplications are reported at the bottom.

References

1. Musante, L.; Faletra, F.; Meier, K.; Tomoum, H.; Najarzadeh Torbati, P.; Blair, E.; North, S.; Gärtner, J.; Diegmann, S.; Beiraghi Toosi, M.; et al. TTC5 Syndrome: Clinical and Molecular Spectrum of a Severe and Recognizable Condition. *Am J Med Genet A* **2022**, *188*, 2652–2665, doi:10.1002/ajmg.a.62852.
2. Highnam, G.; Wang, J.J.; Kusler, D.; Zook, J.; Vijayan, V.; Leibovich, N.; Mittelman, D. An Analytical Framework for Optimizing Variant Discovery from Personal Genomes. *Nat Commun* **2015**, *6*, 6275, doi:10.1038/ncomms7275.
3. Richards, S.; Aziz, N.; Bale, S.; Bick, D.; Das, S.; Gastier-Foster, J.; Grody, W.W.; Hegde, M.; Lyon, E.; Spector, E.; et al. Standards and Guidelines for the Interpretation of Sequence Variants: A Joint Consensus Recommendation of the American College of Medical Genetics and Genomics and the Association for Molecular Pathology. *Genet Med* **2015**, *17*, 405–424, doi:10.1038/gim.2015.30.
4. Limongelli, I.; Marini, S.; Bellazzi, R. PaPI: Pseudo Amino Acid Composition to Score Human Protein-Coding Variants. *BMC Bioinformatics* **2015**, *16*, 123, doi:10.1186/s12859-015-0554-8.
5. Adzhubei, I.; Jordan, D.M.; Sunyaev, S.R. Predicting Functional Effect of Human Missense Mutations Using PolyPhen-2. *Curr Protoc Hum Genet* **2013**, *Chapter 7*, Unit7.20, doi:10.1002/0471142905.hg0720s76.
6. Sim, N.-L.; Kumar, P.; Hu, J.; Henikoff, S.; Schneider, G.; Ng, P.C. SIFT Web Server: Predicting Effects of Amino Acid Substitutions on Proteins. *Nucleic Acids Res* **2012**, *40*, W452-457, doi:10.1093/nar/gks539.
7. Jian, X.; Boerwinkle, E.; Liu, X. In Silico Prediction of Splice-Altering Single Nucleotide Variants in the Human Genome. *Nucleic Acids Res* **2014**, *42*, 13534–13544, doi:10.1093/nar/gku1206.
8. Kircher, M.; Witten, D.M.; Jain, P.; O'Roak, B.J.; Cooper, G.M.; Shendure, J. A General Framework for Estimating the Relative Pathogenicity of Human Genetic Variants. *Nat Genet* **2014**, *46*, 310–315, doi:10.1038/ng.2892.
9. Schwarz, J.M.; Rödelsperger, C.; Schuelke, M.; Seelow, D. MutationTaster Evaluates Disease-Causing Potential of Sequence Alterations. *Nat Methods* **2010**, *7*, 575–576, doi:10.1038/nmeth0810-575.

10. Bayat, A.; Fenger, C.D.; Techlo, T.R.; Højte, A.F.; Nørgaard, I.; Hansen, T.F.; Rubboli, G.; Møller, R.S. Impact of Genetic Testing on Therapeutic Decision-Making in Childhood-Onset Epilepsies—a Study in a Tertiary Epilepsy Center. *Neurotherapeutics* **2022**, *19*, 1353–1367, doi:10.1007/s13311-022-01264-1.
11. Lefebvre, M.; Bruel, A.-L.; Tisserant, E.; Bourgon, N.; Duffourd, Y.; Collardeau-Frachon, S.; Attie-Bitach, T.; Kuentz, P.; Assoum, M.; Schaefer, E.; et al. Genotype-First in a Cohort of 95 Fetuses with Multiple Congenital Abnormalities: When Exome Sequencing Reveals Unexpected Fetal Phenotype-Genotype Correlations. *J Med Genet* **2021**, *58*, 400–413, doi:10.1136/jmedgenet-2020-106867.
